# MicroRNA-510 promotes cell and tumor growth by targeting peroxiredoxin1 in breast cancer

**DOI:** 10.1186/bcr3464

**Published:** 2013-08-23

**Authors:** Qi J Guo, Jamie N Mills, Savannah G Bandurraga, Lourdes M Nogueira, Natalie J Mason, E Ramsay Camp, Amanda C Larue, David P Turner, Victoria J Findlay

**Affiliations:** 1 Department of Pathology & Laboratory Medicine, 39 Sabin Street, Medical University of South Carolina, Charleston, SC 29425, USA; 2 Department of Surgery, 25 Courtenay Drive, Medical University of South Carolina, Charleston, SC 29425, USA

**Keywords:** MicroRNA, peroxiredoxin1, tumorigenesis, breast cancer, migration, miR-510

## Abstract

**Introduction:**

MicroRNAs are small non-coding RNAs that are involved in the post-transcriptional negative regulation of mRNAs. MicroRNA 510 (miR-510) was initially shown to have a potential oncogenic role in breast cancer by the observation of its elevated levels in human breast tumor samples when compared to matched non-tumor samples. Few targets have been identified for miR-510. However, as microRNAs function through the negative regulation of their direct targets, the identification of those targets is critical for the understanding of their functional role in breast cancer.

**Methods:**

Breast cancer cell lines were transfected with pre-miR-510 or antisense miR-510 and western blotting and quantitative real time PCR were performed. Functional assays performed included cell growth, migration, invasion, colony formation, cytotoxicity and *in vivo *tumor growth. We performed a PCR assay to identify novel direct targets of miR-510. The study focused on peroxiredoxin 1 (PRDX1) as it was identified through our screen and was bioinformatically predicted to contain a miR-510 seed site in its 3' untranslated region (3'UTR). Luciferase reporter assays and site-directed mutagenesis were performed to confirm PRDX1 as a direct target. The Student's two-sided, paired *t-*test was used and a *P*-value less than 0.05 was considered significant.

**Results:**

We show that miR-510 overexpression in non-transformed and breast cancer cells can increase their cell growth, migration, invasion and colony formation *in vitro*. We also observed increased tumor growth when miR-510 was overexpressed *in vivo*. We identified PRDX1 through a novel PCR screen and confirmed it as a direct target using luciferase reporter assays. The reintroduction of PRDX1 into breast cancer cell lines without its regulatory 3'UTR confirmed that miR-510 was mediating its migratory phenotype at least in part through the negative regulation of PRDX1. Furthermore, the PI3K/Akt pathway was identified as a positive regulator of miR-510 both *in vitro *and *in vivo*.

**Conclusions:**

In this study, we provide evidence to support a role for miR-510 as a novel oncomir. We show that miR-510 directly binds to the 3'UTR of PRDX1 and blocks its protein expression, thereby suppressing migration of human breast cancer cells. Taken together, these data support a pivotal role for miR-510 in breast cancer progression and suggest it as a potential therapeutic target in breast cancer patients.

## Introduction

Breast cancer is the most common cancer in women worldwide, resulting in 350,000 deaths each year [1,2]. Most deaths due to breast cancer are the result of metastasis, demonstrated by the drop in five-year survival from 90% to just 23% in women presenting with metastatic disease [3]. Metastasis involves epithelial-to-mesenchymal transition (EMT) and cellular changes leading to a more invasive phenotype. These invasive changes are critical steps in breast cancer progression and can lead to treatment failure [4]. A better understanding of the mechanisms underlying these phenotypic changes will allow improved prediction of those patients susceptible to metastasis as well as improved therapeutic strategies [1].

Previous studies have suggested a role for microRNAs in regulation of metastasis, invasion, proliferation, cell cycle, growth, differentiation and apoptosis [5-8]. MicroRNAs (miRNAs) are small, non-coding RNA molecules approximately 18 to 25 nucleotides in length [9]. They comprise approximately 3% of the human genome and regulate approximately 30% of transcripts [10,11]. Approximately half of miRNAs have been found in "fragile sites", regions associated with cancer [10]. miRNAs negatively regulate expression of target genes by binding to the 3'UTR of mRNA transcripts to either cause degradation or prevent translation, depending upon complementarity [5,9]. miRNAs can regulate expression of many different types of genes and have been shown to function as both tumor suppressors and oncogenes [5,6,12].

Calin *et al*. [13] were the first to show involvement of aberrant miRNA expression in cancer progression. Since then, many studies have demonstrated that dysregulation of miRNAs have implications in invasion, migration and metastasis in breast cancer [7,14,15]. Our studies have shown that miRNA 510 (miR-510), is elevated in breast tumor samples while absent in the matched non-tumor breast tissue samples [15]. These studies identify Peroxiredoxin 1 (PRDX1) as a novel direct target of miR-510. PRDX1 is a member of a family of peroxidases with six isoforms known to be involved in protection of cells against oxidative stress [16,17]. Deletion of PRDX1 has been shown to promote tumor growth in mice [18]. It is ubiquitously and highly expressed and functions as a tumor suppressor [18,19]. The goal of this study was to investigate the role of miR-510 in breast cancer cell migration and tumor growth and to verify PRDX1 as the direct miR-510 target underlying the mechanism of these phenotypic changes.

## Materials and methods

### Cell culture and reagents

Human breast cancer cell lines (MCF7, CAMA-1, MDA-MB-231, MCF10A and BT549) were cultured and maintained at 37°C with 5% CO_2 _in medium supplemented with 10% fetal bovine serum and 100 U of penicillin/streptomycin. MCF7, CAMA-1, MDA MB 231 and HEK293 cells were grown in DMEM media. BT549 cells were grown in RPMI media. MCF10A cells were grown in DMEM:F12 (50:50) media. MCF7 media was supplemented with 1 mM sodium pyruvate, 1 mM sodium bicarbonate, 2 mM L-glutamine, 0.1 mM nonessential amino acids and 0.01 mg/mL insulin. MCF10A media was supplemented with 2 mM L-glutamine, 5% horse serum, 10 μg/mL insulin, 20 ng/mL epidermal growth factor (EGF), 500 ng/mL hydrocortisone, and 10 μg/mL cholera toxin. The breast cancer cell line CAMA-1 was a kind gift of R. Neve (University of California, San Francisco, CA, USA). All other lines were obtained from ATCC (Manassas, VA, USA). Ethical approval for our work with human breast cancer cell lines was not required for our *in vitro *studies. All tissue culture reagents were purchased from Invitrogen (Carlsbad, CA, USA). shPrdx1 vectors were obtained from the Hollings Cancer Center shRNA core laboratory (Medical University of South Carolina, Charleston, SC, USA).

### Immunohistochemistry

Antigen retrieval was done by heating in a microwave oven for 2 × 3 minutes on 30% power in 10 mmol/L citrate (pH 6.0), followed by 30 minutes in a steamer. Sections were washed, treated with 0.3% H_2_O_2 _for 30 minutes and non-specific binding was blocked with 2.5% horse serum (ImmPRESS Vector staining kit; Vector Laboratories, Burlington, CA, USA) for 20 minutes and then incubated overnight at 4°C with Ki67 or p-Akt primary antibody at a 1:200 and 1:50 dilution, respectively, in 2.5% normal horse serum in PBS. Overnight incubation at 4°C was followed by 3 × 10-minute washes in PBS, Immpress anti-rabbit secondary antibody was incubated (Vector Laboratories) for 30 minutes at room temperature. After washing with H_2_O, 3,3'-diaminobenzidine substrate (Sigma, St Louis, MO, USA) was added for two minutes followed by washing in H_2_O. Slides were counterstained with hematoxylin.

### Quantitative reverse transcription PCR

Total RNA from cancer cell lines was extracted using the RNeasyPlus Mini Kit (Qiagen, Valencia, CA, USA). Total RNA measuring 1 μg was reverse transcribed in a 20 μl reaction using iScript (Bio-Rad, Hercules, CA, USA). Real time PCR for gene expression was performed with 5 μl of a 1:20 dilution of reverse transcribed cDNA using the universal probe library (UPL) system (Roche, Nutley, NJ, USA) in a LightCycler 480 (Roche). The cycling conditions were performed as per the manufacturer's instructions. Primer sequences for PRDX1 were: forward 5'-cactgacaaacatggggaagt-3' and reverse 5'-tttgctcttttggacatcagg-3' together with UPL probe #20; and for Akt1 forward 5'- gcagcacgtgtacgagaaga-3' and reverse 5'-ggtgtcagtctccgacgtg-3' together with UPL probe #45. Triplicate reactions were run for each cDNA sample. The relative expression of each gene was quantified on the basis of Ct value measured against an internal standard curve for each specific set of primers using the software provided by the instrument manufacturer (Roche). These data were normalized to GAPDH using the primer sequences: forward 5'-agccacatcgctcagacac-3' and reverse 5'-gcccaatacgaccaaatcc-3' together with UPL probe #60.

### Taqman analysis

For microRNA analysis RNA was extracted as described above using the RNeasyPlus Mini Kit from Qiagen. Total RNA measuring 100 ng was reverse transcribed using miR-510 specific primers using the Applied Biosystems (Grand Island, NY, USA) reverse transcription kit as per the manufacturer's instructions. Real time PCR was performed with 1 μl of reverse transcribed cDNA using the TaqMan Assay from Applied Biosystems as per the manufacturer's instructions on the Roche LightCycler 480.

### Generation of stable cell lines

The cloning of miR-510 into pSuppressor-neo vector is already described [15]. For the generation of clonal stable MCF10A cells overexpressing miR-510 (510-1; 510-10; 510-11), pSuppressor-neo vector (Imgenex, San Diego, CA, USA) expressing miR-510 was transfected into MCF10A cells and stable cells were selected in medium containing G418. The wild type 3'UTR of PRDX1 was cloned into the *Xba*I site of the pGL3-promoter vector (Promega, Madison, WI, USA) using the primers PRDX1_3UTRf 5'-gcgctctagagcgctgggctgt-3' and PRDX1_3UTRr 5'-gcgctctagagactcatcaaggtctcagt-3'. The sequence complementary to the seed of miR-510 was deleted with the primers PRDX1mutF 5'-ttggtaggaatggcctggcgttgtgggcag-3' and PRDX1mutR 5'-ctgcccacaacgccaggccattcctaccaa-3' using a QuikChange Site-Directed Mutagenesis Kit (Stratagene, La Jolla, CA, USA). All constructs were validated by sequencing at MWG Operon (Huntsville, AL, USA).

### Lentiviral stable pools

Stable expression of miR-510 in MCF10A, MCF7 and MDA MB 231 cells was achieved through lentiviral infection. Stable expression was achieved through selection in puromycin (Invitrogen). Lentiviral miR-510 and control vectors (pEZX) were purchased directly from GeneCopoeia (Rockville, MD, USA) and lentiviral preparations were made using the Maine Medical Center Research Institute cell culture and viral vector core (Scarborough, ME, USA).

### Oligonucleotide transfection

The miRNA inhibitors (Ambion, Austin, TX, USA) are single-stranded chemically enhanced oligoribonucleotides designed to inhibit the endogenous miRNAs. Cells were transfected with the indicated amounts of oligoribonucleotide using the XtremeGene siRNA reagent as per the manufacturer's instructions (Roche). A total of 48 or 72 h after transfection, cells were harvested for protein or RNA extraction and/or assay.

### Plasmid transfection

Transient transfections were performed with the indicated amounts of vector using the XtremeGene HP reagent as per the manufacturer's instructions (Roche). A total of 48 or 72 hours after transfection, cells were harvested for protein or RNA extraction and/or assay.

### Luciferase assays

Cells were plated at 50,000 cells per well in a 24-well plate. The pGL3 reporter constructs (0.5 μg, firefly luciferase) were co-transfected with pRL-TK (0.05 μg, *Renilla *luciferase) using NanoJuice as per the manufacturer's instructions (Novagen, Gibbstown, NJ, USA). Luciferase activity was measured after 48 h using the dual luciferase reporter assay system (Promega). Firefly luciferase activity was normalized to *Renilla *luciferase activity for each transfected well.

### Western blot analysis

Cell lysate preparation and Western blot analysis using enhanced chemiluminescence were performed as described previously [15]. Experimental antibodies include human PRDX1 (Abcam, Cambridge, MA, USA). GAPDH and beta-actin (Abcam) were used as loading controls.

### Pacman (haptokinetic) migration track assay

Wells within a two-well chamber slide were pre-coated with 5 μg/mL fibronectin and then overlaid with a field of 1 μm in diameter carboxylate-modified polystyrene fluorescent microspheres (Invitrogen). Cells were then seeded at low density (approximately 4/mm^2^) in normal growth medium and incubated for a period of 24 h. The ability of the cells to create nonfluorescent tracks was then assessed by fluorescent microscopy and quantified using NIH image. Error bars represent the SD from 10 migration tracks in three separate experiments.

### Transwell migration and invasion assay

Cells were seeded into the upper chamber of a Transwell insert pre-coated with 5 μg/ml fibronectin for migration or a BD™Matrigel invasion chamber for invasion, in serum-free medium at a density of 50,000 cells per well (24-well insert; pore size, 8 μM; BD Biosciences, San Jose, CA, USA). Medium containing 10% serum was placed in the lower chamber to act as a chemo-attractant, and cells were further incubated for 4 h (migration) and 24 h (invasion). Non-migratory cells were removed from the upper chamber by scraping with a cotton bud. The cells remaining on the lower surface of the insert were stained using Diff-Quick (Dade Behring, Inc., Newark, DE, USA). Cells were quantified as the number of cells found in five random microscope fields in two independent inserts. Error bars represent the SD from three separate experiments.

### Colony formation assay

Wild-type and miR-510 stably transformed MCF10A cells were seeded at a cell density of approximately 4 cells/mm^2 ^in normal growth media. Cells were incubated as normal, and colonies were counted after 7 to 10 d.

### Cell growth assay

Cell growth was measured using the SRB assay [20]. Cells were plated into each well of a 96- well plate and cells were fixed at the indicated time points with ice-cold 5% trichloroacetic acid (TCA), washed and stained with sulforhodamine B (SRB) and the optical density was measured at 560 nm.

### Trypan blue/cell viability assay

Cells were either untreated or treated with 50 μM for 24 hours. Cells were collected by trypsinization and 20 μl was mixed 1:1 with trypan blue and counted on an automated cell counter.

### Tumor growth

A total of 1 × 10^6 ^MDA-MB-231 cells stably transfected with either miR-510 or scramble control were injected orthotopically into eight-week-old female nude mice. Tumors were measured biweekly with electronic calipers and tumor volume calculated using the formula (L × W^2^)/2.

### In vivo protocol approval

Research protocols were designed and conducted in accordance with the guidelines set by the Institutional Animal Care and Use Committee, Medical University of South Carolina, Approval # ARC-2907.

### Statistical analysis

For statistical testing, two-sided paired Student's *t*-tests were done using an Excel spreadsheet. *P-*values are given for each individual experiment, but in general, *P *< 0.05 was considered statistically significant. Error bars represent standard deviations of three independent experiments unless indicated otherwise.

## Results

### MicroRNA 510 promotes proliferation, migration, invasion and colony formation in vitro

**Figure 1:**
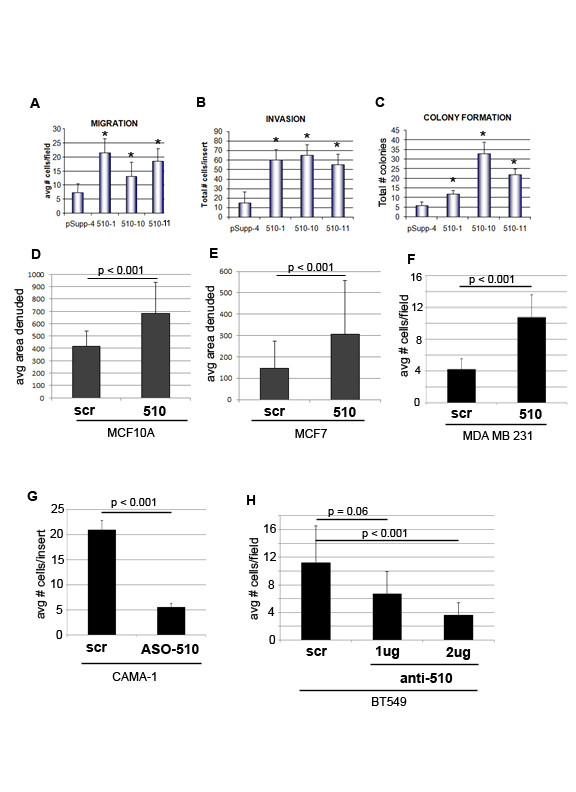
**MicroRNA 510 promotes migration, invasion and colony formation *in vitro***. Transwell migration (**A**), matrigel invasion (**B**) and colony formation (**C**) assays of 3 independent stable clones over-expressing miR-510 (510-1, 510-10 and 510-11) compared to vector control (pSupp-4). Quantitation of haptokinetic migration (Pacman) assay of MCF10A (**D**) and MCF7 (**E**) cells stably infected with miR-510 (510) compared to stable infected scrambled control (scr). (**F**) Transwell migration assay of MDA MB 231 cells stably infected with miR-510 (510) compared to stable infected scrambled control (scr). (**G**) Transwell migration assay of CAMA-1 cells transfected with antisense oligonucleoteide against miR-510 (ASO-510) or scrambled control (scr). (**H**) Transwell migration assay of BT549 cells transfected with increasing concentrations of anti-miR-510 vector (anti-510) or scrambled vector control (scr). *P < 0.05.

**Figure 2:**
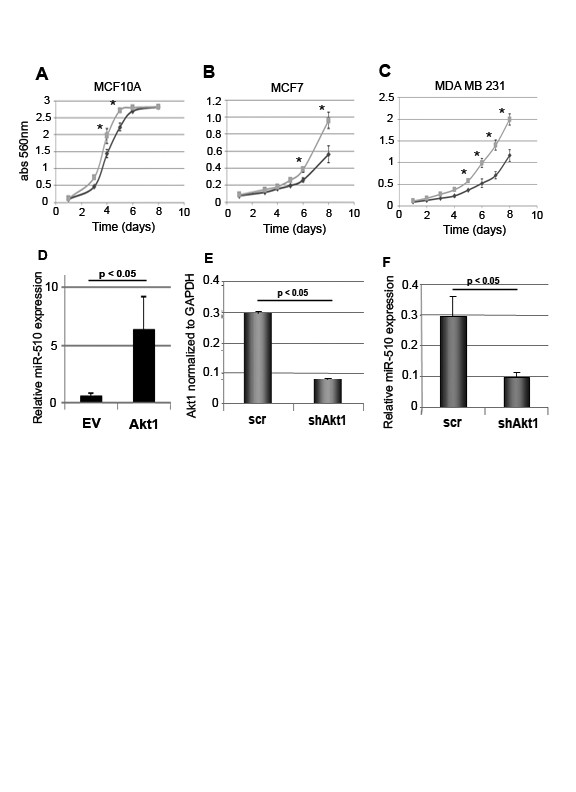
**MicroRNA 510 increases cellular proliferation *in vitro***. Cellular proliferation of stable infected miR-510 (light gray lines) MCF10A (**A**), MCF7 (**B**) and MDA MB 231 (**C**) cell lines compared to stable infected scrambled control (dark gray lines). (**D**) qPCR of miR-510 levels in MCF10A cells transiently transfected with Akt1 or vector alone (EV). qPCR of Akt1 (**E**) and miR-510 (**F**) levels in MDA-MB-175 VII breast cancer cells transduced with short hairpin Akt1 (shAkt) or scrambled control (scr). *P < 0.05.

Our previous studies have shown that transient miR-510 expression increases the migration and invasion of non-invasive MCF7 breast cancer cells [15]. However, the functional effects of miR-510 expression in non-transformed breast cells have not been assessed. To assess whether migration and/or invasion are altered in miR-510 overexpressing MCF10A cells, we performed transwell migration assays across a chemokine gradient, and invasion assays through Matrigel. The number of cells found to migrate or invade in miR-510 overexpressing cells was significantly increased compared with the parental control. Using three mir-510 independent clones we observe a two- to four-fold increase in migration (Figure 1A) and four- to five-fold increase in invasion (Figure 1B) across coated membranes. To further explore the contribution of miR-510 to cancer progression, we investigated the ability of the stably transfected MCF10A cells to proliferate when seeded at low density *in vitro *using a clonogenic assay and observed an increase in the total number of colonies formed when compared with the parental control (Figure 1C). Colony formation was increased two- to six-fold in the mir-510 stably expressing clones. To assess the functional effects of miR-510 when expressed at more physiological levels, we established stable breast cell lines expressing miR-510 after lentivirus transduction. These included the non-transformed MCF10A cells, the non-invasive MCF7 and the invasive MDA MB 231 cancer cells. Proliferation and migration are two major cellular processes that are required for tumor growth and metastasis. Therefore, to assess the role of miR-510 in the regulation of these important processes, we first measured the ability of miR-510 to increase migration in 1) non-transformed breast (MCF10A), 2) non-invasive breast cancer (MCF7) and 3) invasive breast cancer (MDA MB 231) cells. miR-510 expression was able to increase the ability of MCF10A cells (Figure 1D) and MCF7 cells (Figure 1E) to migrate in the absence of a stimuli as assessed by the Pacman haptokinetic migration track assay (Additional file 1, Figure S1). Similarly, miR-510 was able to increase the migration of MDA MB 231 cells (Figure 1F) in the presence of a stimulus as assessed by transwell migration assay. Reciprocal to this we found that inhibition of miR-510 by transfection with either antisense oligonucleotides (ASO-510) or anti-miR510 vector (anti-510) in the breast cancer cell lines CAMA-1 (Figure 1G) and BT549 (Figure 1H) was able to inhibit migration in the presence of a stimuli as assessed by transwell migration assay. Proliferation was assessed in breast cell lines that were stably infected with miR-510 over a course of seven days (Figure 2) and we observed a significant increase in the rate of cellular proliferation in MCF10A (Figure 2A), MCF7 (Figure 2B) and MDA MB 231 (Figure 2C) miR-510 expressing cells when compared to the scramble controls.

### MicroRNA 510 expression is regulated by the AKT Pathway

To determine which signaling pathways were involved in the activation of miR-510 we performed a luciferase reporter screening assay utilizing a construct that contains a functional miR-510 seed sequence site [15]. We treated the cells with a panel of kinase inhibitors and measured luciferase expression compared to untreated controls. We observed an increase in luciferase activity in the cells treated with the PI3K inhibitor LY294002 when compared to the untreated control, suggesting that the PI3K/Akt pathway might be involved in the activation of miR-510 expression (data not shown). To test this hypothesis, we transiently overexpressed Akt1 in MCF10A cells and performed real time PCR to assess miR-510 expression levels (Figure 2D). We observed a significant (approximately seven-fold) increase in miR-510 levels in MCF10A cells overexpressing Akt1 compared to the empty vector control. To examine the role of Akt1 in the regulation of miR-510 expression we transfected MDA MB 175 VII cells, which express miR-510 at relatively high levels, with a short hairpin vector targeting Akt1 (shAkt1; Figure 2E, F). We performed real time PCR to assess miR-510 expression levels (Figure 2F) and observed a decrease in the levels of miR-510 in the shAkt1 transduced cells when compared to the scrambled control. Taken together these data suggest that the PI3K/Akt pathway may function in the activation of miR-510 in breast cancer.

### MicroRNA 510 promotes tumor growth *in vivo*

**Figure 3:**
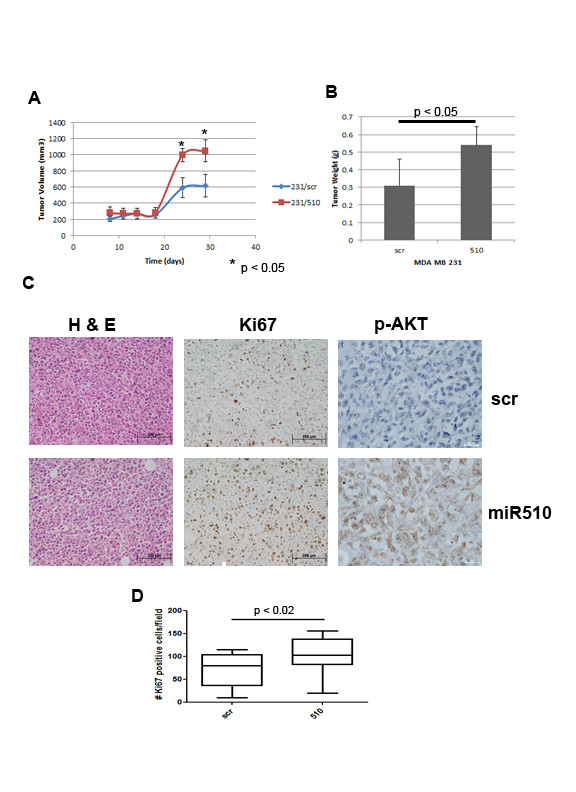
**MicroRNA 510 increases tumor growth *in vivo***. Panels show tumor volume (**A**), tumor weight (**B**), Immunohistochemistry (**C**) and Ki67 quantitation (**D**) of tumors resulting from orthotopic injection of miR-510 (510) or scrambled control (scr) stably infected MDA MB 231 cells into female nude mice.

To assess the functional effects of miR-510 *in vivo*, we injected nude mice orthotopically with MDA MB 231 cells stably infected with miR-510 or scramble control as described above. Tumor growth was monitored twice weekly for 30 days. Tumors from both miR-510 expressing and scrambled controls appeared to initiate at a similar time frame. However, by three weeks the miR-510 expressing tumors were growing more rapidly and by the end of the study, the miR-510 expressing tumors were larger (tumor volume) and heavier (tumor weight) than the scrambled controls (Figure 3A, B). Histologically, the tumors looked similar; however, Ki67 staining showed that the miR-510 expressing tumors were more proliferative than the scrambled controls (Figure 3C, D). Based on our *in vitro *observations with the PI3K/Akt pathway and miR-510 expression, we performed IHC with p-Akt and observed an increased level of phosphorylated or active Akt in tumors expressing miR-510 (Figure 3C).

### Peroxiredoxin 1 is a direct target of miR-510

**Figure 4:**
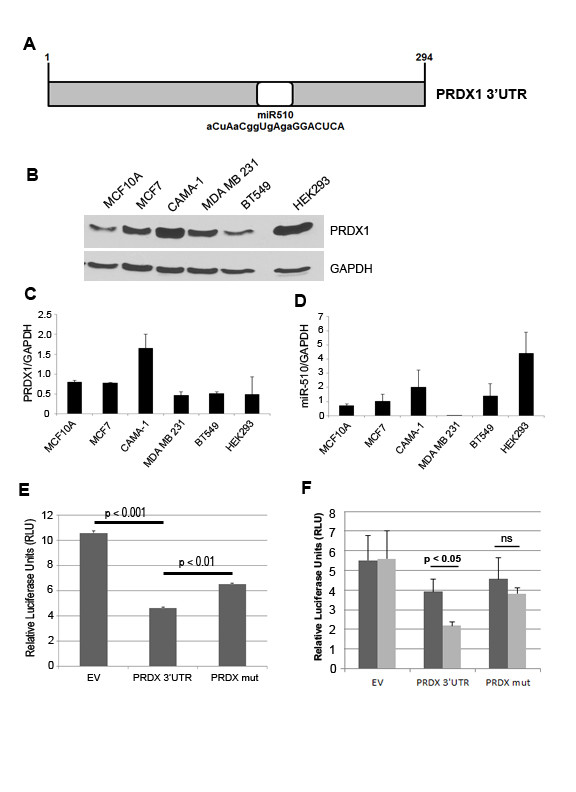
**PRDX1 is a direct target of microRNA 510**. (**A**) Schematic representation of the predicted target site of miR-510 in the 3'UTR of peroxiredoxin 1 (PRDX1) mRNA. The numbers 1 to 294 represent base pairs in the 3'UTR of PRDX1. Complementary base sequences are highlighted in upper case letters. Western blot (**B**) and quantitative PCR (**C **and **D**) analysis of endogenous PRDX1 (B and C) and miR-510 (D) levels in the cell lines used in the study. GAPDH is shown as a loading control for western blot in B and is used for normalization in C and D. (**E**) Luciferase activity of HEK293 cells transfected with pGL3 promoter luciferase reporter vector alone (EV), the PRDX1 3'UTR reporter construct (PRDX 3'UTR) or PRDX1 3'UTR reporter construct mutated in the miR510 seed sequence binding site (PRDX mut). (**F**) Luciferase activity of MDA MB 231 cells stably transfected with scrambled control (dark gray bars) or miR-510 (light gray bars) transiently transfected with reporter constructs described in (E). All luciferase assays were normalized to Renilla luciferase activity. The data are expressed as the mean ± SD for three experiments conducted in triplicate.

A PCR-based screen [21] was performed in order to identify novel targets of miR-510, as microRNAs are mainly thought to exert their effects through their negative regulation and direct binding to the 3'UTR of their target mRNA. Peroxiredoxin 1 (Prdx1) is bioinformatically predicted and was identified through our screen to be a direct target of miR-510 (4A, Additional file 2, Table S1). We performed Western blot (Figure 4B) and qPCR (Figure 4C, D) to assess the levels of endogenous PRDX1 and miR-510 levels in the various breast cell lines used in the study. Prdx1 is an abundant antioxidant protein and we found that it was present in all cell lines examined (Figure 4B), but found no direct correlation between miR-510 and Prdx1 levels in the cell lines we examined (Figure 4B, D). To validate Prdx1 as a miR-510 target, and to examine whether Prdx1 expression is repressed by miR-510 through the predicted elements, a luciferase reporter construct containing the 3' UTR of Prdx1 was transfected into HEK293 cells (Figure 4E). The presence of the 3'UTR of Prdx1 (PRDX 3'UTR) led to a decrease in luciferase expression when compared to the empty vector (EV) control. The most important criteria for target recognition are the 5' five to eight nucleotide core sequence of a miRNA, known as the "seed sequence." To further validate that the predicted miR-510 seed sequence within the PRDX1 3'UTR was functional, we mutated the seed sequence of miR-510 in the luciferase reporter construct. Transfection of HEK293 cells with the mutated luciferase reporter construct (PRDX mut) resulted in an increase in luciferase activity when compared to the WT Prdx1 3'UTR (Figure 4E). Mutation of the seed sequence of miR-510 within the 3'UTR did not fully restore luciferase activity to the levels observed in the cells transfected with no 3'UTR construct, suggesting that other miRNA binding sites are present and functional within the 3'UTR of PRDX1. To determine whether the PRDX1 3'UTR was responsive to miR-510 expression we transfected MDA MB 231 cells stably infected with miR-510 or scrambled controls with the luciferase reporter vectors (Figure 4F). We observed an increased repression of the PRDX1 3'UTR when miR-510 was expressed when compared to the scrambled controls. However, no further significant decrease in luciferase activity was observed in either the cells transfected with the PRDX mut construct or the EV control suggesting that miR-510 directly binds to the predicted site within the 3'UTR of PRDX1 to negatively regulate its expression.

**Figure 5:**
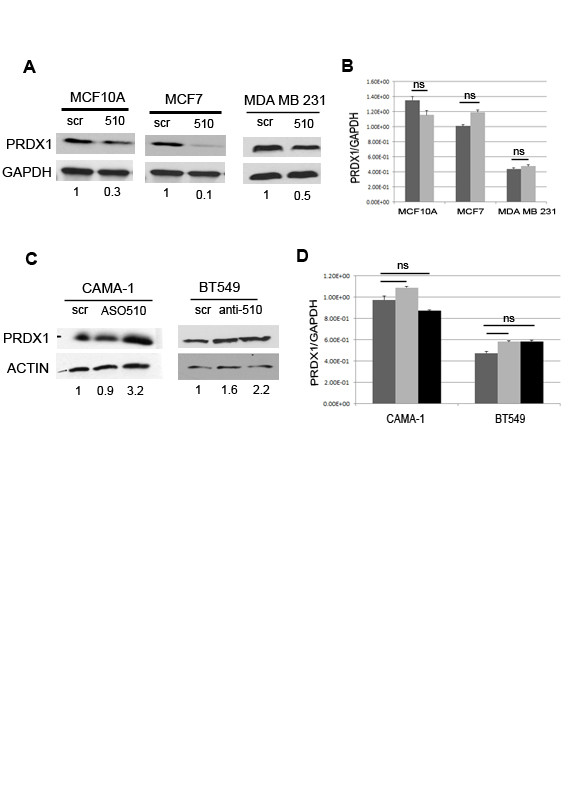
**miR-510 regulates endogenous PRDX1 protein expression**. (**A**) Western blot and (**B**) quantitative PCR analysis of endogenous peroxiredoxin 1 (PRDX1) expression in MCF10A, MCF7 and MDA MB 231 cells stably infected with scrambled control (scr) or miR-510 (510). (A) GAPDH is shown as a loading control. (B) Relative expression is normalized to GAPDH; scr - dark gray bars, miR-510 - light gray bars. (**C**) Western blot and (**D**) quantitative PCR analysis of CAMA-1 and BT549 cells transiently transfected with increasing concentrations of antisense oligonucleotides to miR-510(ASO-510) or antimiR-510 vector (anti-510). (**C**) ACTIN is shown for as a loading control. (**D**) Relative expression is normalized to GAPDH; scr - dark gray bars, ASO-510 (150 nM) anti miR-510 (1 ug) - light gray bars, ASO-510 (450 nM) anti miR-510 (2 ug)- black bars). Fold change in PRDX1 protein levels compared to scr control were quantified and are shown under each Western blot image.

To assess the effects of miR-510 on endogenous Prdx1 expression, both protein and RNA levels were assessed in MCF10A, MCF7 and MDA MB 231 cells. We observed a decrease in the levels of Prdx1 protein in all of the cells tested when miR-510 was expressed, whereas the levels of Prdx1 mRNA were relatively unchanged, suggesting that miR-510 regulates Prdx1 through translational repression as opposed to mRNA degradation (Figure 5A, B). CAMA-1 and BT549 breast cancer cells were used as a model system to look at loss of function as they express miR-510 at the highest levels of all the breast lines examined. Transfection of antisense oligoribonucleotides (ASO) targeted against miR-510 (ASO-510) in CAMA-1 cells and anti-miR510 vector (anti-510) in BT549 cells resulted in an increase in Prdx1 protein levels, whereas the Prdx1 mRNA levels remained unchanged (Figure 5C, D).

### Prdx1 expression inhibits miR-510-mediated cell migration

**Figure 6:**
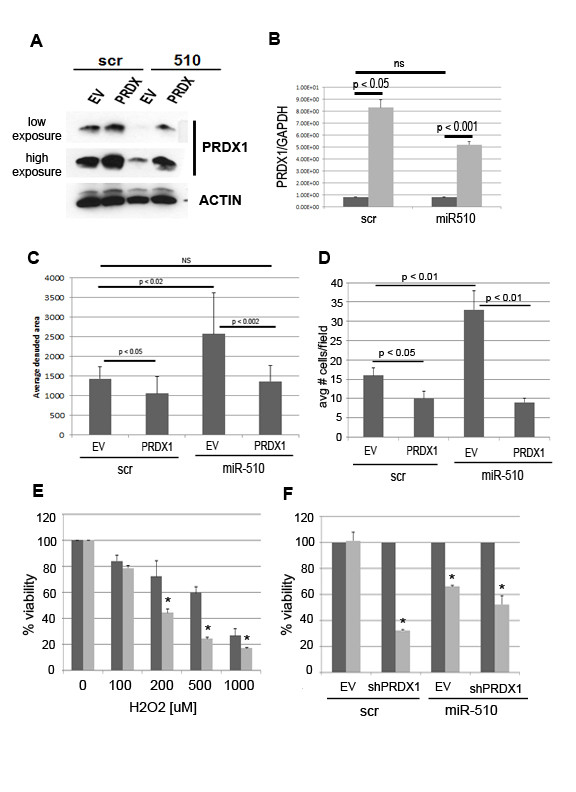
**Functional effects of miR-510 mediated negative regulation of PRDX1**. Western blot of PRDX1 protein expression (**A**), quantitative real time PCR of peroxiredoxin 1 (PRDX1) mRNA levels (**B**) and quantified Pacman haptokinetic migration track assays (**C**) of miR-510 (510) or scrambled control (scr) MCF10A cells transiently transfected with PRDX1 ORF (PRDX1; light gray bars in B) or empty vector (EV; dark gray bars in B). Western blots are normalized to ACTIN and real time PCR to GAPDH. A low and high exposure of the same PRDX1 blot is shown as indicated. Quantitation of haptokinetic migration track assays is of 10 independent tracks per experiment. (**D**) Transwell migration assay of MCF7 cells stably infected with miR-510 or scrambled (scr) control transiently transfected with PRDX1 or empty vector (EV). (**E**) Trypan blue exclusion assay of miR-510 expressing (light gray bars) or scrambled control (dark gray bars) MCF10A cells treated with increasing concentrations of hydrogen peroxide (H_2_O_2_) for 24 h. (**F**) Trypan blue exclusion assay of miR-510 expressing or scrambled control (scr) MDA MB 231 cells transiently transfected with shPRDX1 vector or empty vector (EV) control for 48 h before treatment with 0 (dark gray bars) and 50 μM (light gray bars) H_2_O_2 _for 24 h. Data in E and F are plotted as percent live cells compared to untreated control. (E) *P < 0.05 compared to scr control. (F) *P < 0.05 compared to untreated control.

miRNAs have multiple targets and, therefore, the effects observed after miR-510 expression may be the result of the increased Prdx1 protein, as well as non-Prdx1-related miR-510 effects. One way to evaluate these possibilities is to examine the phenotypes in cells in which a non-targeted Prdx1 is expressed. To do this, the ORF of Prdx1 was transfected into the MCF10A and MCF7 miR-510 expressing cells and cellular migration assessed (Figure 6). As we have previously observed, the protein levels of Prdx1 were reduced in the miR-510 expressing cells (Figure 6A). In addition, we also see that Prdx1 protein and mRNA levels are increased in the miR-510 expressing cells when the Prdx1 ORF is transiently overexpressed (Figure 6A, B), suggesting that Prdx1 protein levels are restored to endogenous levels in the miR-510 expressing cells. We observed a small but significant decrease in migration when PRDX1 was overexpressed in scrambled control cells as well as a restoration of migration to control levels in miR-510 expressing cells when PRDX1 was co-expressed in both MCF10A and MCF7 cells (Figure 6C, D).

### miR-510 affects the redox function of Prdx1

Prdx1 is an antioxidant protein and, therefore, its primary function within the cell is involved with the regulation of cellular redox response. To assess whether the miR-510 mediated negative regulation of PRDX1 was able to interfere with this primary function, we performed cell viability assay after treatment with H_2_O_2 _(Figure 6E, F). MCF10A cells stably infected with miR-510 showed increased sensitivity to treatment with H_2_O_2 _when compared to the scrambled control MCF10A cells (Figure 6E). To assess whether miR-510 was able to increase sensitivity to H_2_O_2 _to similar levels as when PRDX1 is inhibited we performed cell viability assay with MDA MB 231 cells stably infected with miR-510 or scr control that were transiently transfected with a short hairpin functionally validated to target PRDX1. After 48 h, cells were either untreated or treated with 50 mM H_2_O_2 _for 24 h. Cell viability was assessed using the trypan blue exclusion assay. Control cells were resistant to this concentration of H_2_O_2 _and showed no cell death. However, transfection with the shPRDX1 led to an increase in sensitivity to H_2_O_2_, and we observed a 60 to 70% increase in cell death. Similarly cells overexpressing miR-510 showed an increased sensitivity to H_2_O_2_, showing a 30 to 40% increase in cell death. However, no significant increase in sensitivity to H_2_O_2 _was observed in cells with shPRDX1 and miR-510 expression when compared to either treatment alone.

## Discussion

Since their discovery, microRNAs have been implicated in many steps of cancer development and progression. They have shown potential roles as predictors of treatment outcomes and microRNA profiling of tumors may have the ability to predict prognosis and identify tumor subtypes [5]. The Croce group has shown that miRNAs are aberrantly expressed in human breast cancers and that this expression correlated to multiple features of cancer, including estrogen and progesterone receptor status, stage, and indices of proliferation and invasion [7].

Currently in the literature there are few studies highlighting the role of miR-510. They include its involvement in regulating expression of the serotonin receptor type 3 in enterocytes of colonic mucosa, indicating a role in irritable bowel syndrome [22,23], as well as identifying elevated levels of miR-510 in Regulatory T cells (Tregs) from Type 1 diabetic patients [24]. We have previously published the role of miR-510 in promoting migration, invasion and colony formation in breast cancer cells [15]. We also observed the levels of miR-510 to be elevated in human breast tumor samples [15]. This study supports the role of miR-510 functioning as an "oncomir", causing increased migration, invasion and colony formation of non-transformed breast and non-invasive breast cancer cells *in vitro *and promoting breast tumor growth *in vivo*.

Peroxiredoxin 1 (PRDX1) functions as a tumor suppressor and has a cytoprotective role in breast cells [18,25]. PRDX1 contains a miR-510 seed sequence in its 3'UTR and we have validated PRDX1 as a direct target of miR-510 and have shown how regulation of PRDX1 by miR-510 contributes to the migratory phenotype observed in miR-510 over-expressing cells. Cao *et al*. showed that loss of PRDX1 promotes PTEN oxidation and activation of Akt [16]. Multiple targets of miR-510 are predicted to directly target multiple negative regulators and effectors of the Akt signaling pathway and, therefore, a potential mechanism of miR-510-mediated increase in cell proliferation, migration, invasion and tumor growth could be through hyperactivation of the Akt signaling pathway. Indeed, we show *in vitro *that overexpression of Akt1 leads to an increase in the expression of miR-510 and that inhibition of Akt1 results in a decrease in the expression levels of miR-510. Furthermore, we show *in vivo *that miR-510 expressing tumors have increased activation of the Akt pathway as demonstrated by an increase in Akt phosphorylation, suggesting that a positive feedback loop of this pathway may be occurring in these cells. We have identified a novel role for PRDX1 in the inhibition of migration and demonstrate here that miR-510 mediated negative regulation of Prdx1 is able to inhibit both its role in migration as well as its more well-known role in cellular redox response. However, further investigation of the mechanism of miR-510 mediated negative regulation of PRDX1 is necessary to fully understand their role in tumorigenesis and breast cancer progression.

## Conclusions

MicroRNA 510 is understudied; however, we have strong evidence to support a pivotal role in breast cancer progression. A greater comprehension of the mechanisms involved in miR-510 mediated tumor progression as well as the direct targets mediating these effects are critical to our understanding of its role in cancer. Exploring the role of miR-510 in metastasis may also allow it to be used as a biomarker and predictor of prognosis in patients, providing the next step toward personalized treatment in breast cancer.

## Abbreviations

3'UTR: 3' untranslated region; ASO: antisense oligoribonucleotides; EGF: epidermal growth factor; EMT: epithelial-mesenchymal-transition; EV: empty vector; miR-510: microRNA 510; GAPDH: glyceraldehyde 3-phosphate dehydrogenase; miRNA: microRNA; PI3K: phosphatidylinositide 3-kinase; PRDX1: peroxiredoxin 1; SRB: sulforhodamine B; Tregs: Regulatory T cells; UPL: universal probe library.

## Competing interests

The authors declare that they have no competing interests.

## Authors' contributions

QJG, JNM, SGB, LMN and NJM participated in and performed the *in vivo *and IHC assays. QJG, SGB and LMN performed the luciferase and Western blot assays. QJG, VJF and JNM performed the qPCR assays. NJM performed pathological assessment of tumors. QJG, VJF and DPT performed the functional and rescue experiments. ERC, ACL, DPT and VJF conceived of the study and participated in its design and coordination and, with JNM, drafted the manuscript. All authors read and approved the final manuscript.

## Supplementary Material

Additional file 1 **Figure 1**. Microscopic images of haptokinetic migration (Pacman) assay of MCF10A (**A**) and MCF7 (**B**) cells stably infected with miR-510 compared to stable infected scrambled control (scr). (**C**) Quantitative PCR analysis of miR-510 levels in MCF10A, MCF7 and MDA MB 231 cells stably infected with miR-510 or scrambled controls normalized to GAPDH. *P < 0.005. Click here for file

Additional file 2 **Table 1**. Direct targets of miR-510 identified in PCR screen. Click here for file
