# Supplementary figures and images for: MicroRNA-510 promotes cell and tumor growth by targeting peroxiredoxin1 in breast cancer
Source: Breast Cancer Res. 2013 Aug 23;15(4):R70. doi: 10.1186/bcr3464 (PMC3978419; doi:10.1186/bcr3464)

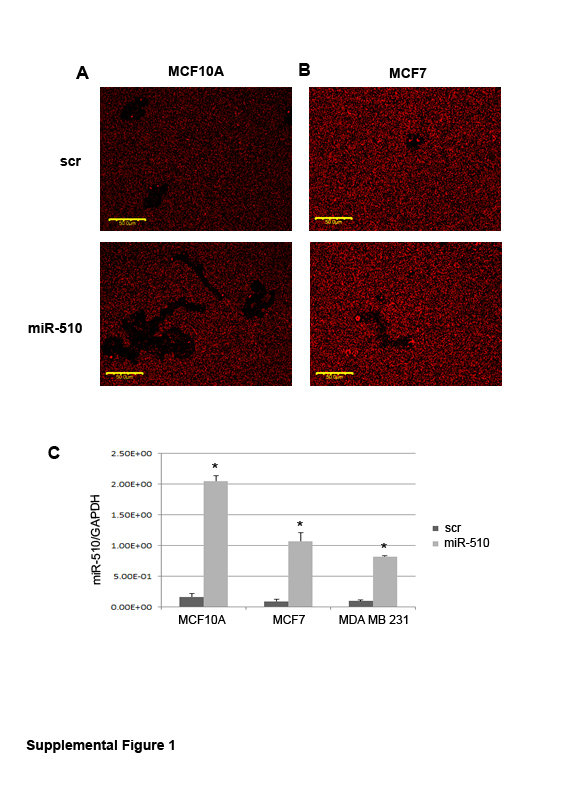

Supplement: Additional file 1 — Figure 1. Microscopic images of haptokinetic migration (Pacman) assay of MCF10A (A) and MCF7 (B) cells stably infected with miR-510 compared to stable infected scrambled control (scr). (C) Quantitative PCR analysis of miR-510 levels in MCF10A, MCF7 and MDA MB 231 cells stably infected with miR-510 or scrambled controls normalized to GAPDH. *P < 0.005. [file bcr3464-S1.jpg]
