# Supplementary material for: MicroRNA-510 promotes cell and tumor growth by targeting peroxiredoxin1 in breast cancer
Source: Breast Cancer Res. 2013 Aug 23;15(4):R70. doi: 10.1186/bcr3464 (PMC3978419; doi:10.1186/bcr3464)
Supplement: Additional file 2 — Table 1. Direct targets of miR-510 identified in PCR screen. [file bcr3464-S2.docx]

| **Gene Symbol** | **Gene Name** | **NCBI RefSeq** |
| --- | --- | --- |
| PRDX1 | Peroxiredoxin 1 | NM_002574 |
| SPDEF | SAM pointed domain containing ets factor | NM_012391 |
| LMO2 | Homo Sapiens LIM domain only 2 (rhombotin-like 1) | NM_005574 |
| POU3F4 | POU class 3 homeobox 4 | NM_000307 |
| RAPGEF2 | Rap guanine nucleotide exchange factor 2 | NM_014247 |
| STAC2 | Human SH3 and cysteine rich domain 2 | NM_198993 |
| EPHA3 | EPH receptor A3 | NM_182644 |
| PIGA | Phosphatidylinositol glycan anchor biosynthesis, class A | NM_020473 |
| INPP5A | Inositol polyphosphate-5-phosphatase, 40kDa | NM_005539 |
| RNA18S5 | RNA, 18S ribosomal 5 | NR_003286 |

**Supplementary Table 1: Direct targets of miR-510 identified in PCR screen**
